# A perspective review on the systematic implementation of ctDNA in phase I clinical trial drug development

**DOI:** 10.1186/s13046-025-03328-4

**Published:** 2025-03-01

**Authors:** Nolwen Guigal-Stephan, Brian Lockhart, Tina Moser, Ellen Heitzer

**Affiliations:** 1 Translational Medicine, Institut de Recherches Servier, 22 route 128, Gif-sur-Yvette, Saclay, 91190 France; 2 Institute of Human Genetics, Diagnostic & Research Center for Molecular BioMedicine, Medical University of Graz, Neue Stiftingtalstrasse 6, Graz, 8010 Austria

**Keywords:** Circulating tumour DNA (ctDNA), Phase I trial, Pharmacodynamics, Pharmacokinetics, Molecular response, RECIST, Tumour fraction

## Abstract

Circulating tumour DNA (ctDNA) represents an increasingly important biomarker for the screening, diagnosis and management of patients in clinical practice in advanced/metastatic disease across multiple cancer types. In this context, ctDNA-based comprehensive genomic profiling is now available for patient management decisions, and several ctDNA-based companion diagnostic assays have been approved by regulatory agencies. However, although the assessment of ctDNA levels in Phase II-III drug development is now gathering momentum, it remains somewhat surprisingly limited in the early Phase I phases in light of the potential opportunities provided by such analysis. In this perspective review, we investigate the potential and hurdles of applying ctDNA testing for the inclusion and monitoring of patients in phase 1 clinical trials. This will enable more informed decisions regarding patient inclusion, dose optimization, and proof-of-mechanism of drug biological activity and molecular response, thereby supporting the evolving oncology drug development paradigm. Furthermore, we will highlight the use of cost-efficient, agnostic genome-wide techniques (such as low-pass whole genome sequencing and fragmentomics) and methylation-based methods to facilitate a more systematic integration of ctDNA in early clinical trial settings.

## Background

Historically, the primary objectives in conventional phase 1 oncology clinical trials focused on safety as well as defining the optimal dose and pharmacokinetics (PK) profile of an investigational drug [1]. However, this model has demonstrated its limitations, particularly in this era of precision-based medicine, and is manifest to some extent by the high attrition rates in early development of oncology drugs where over 90% of investigational drugs in Phase I never achieve market authorization [2]. Although there are many reasons for these low success rates, the major factors driving this attrition are related to toxicity and to an absence of efficacy of the investigational drug. This highlights the need to establish new strategies to enable improved data-driven decisions in early Phase I trials. From a Pharma perspective, terminating developments of poor treatments quickly while enabling accelerated development for highly promising interventions will be a key driver in this evolution. However, one major obstacle to addressing biomarkers of pharmacodynamics (PD) in Phase I is that repeated longitudinal on-treatment tumour biopsies are not feasible, thus pleading for the necessity to implement new approaches to assess molecular response.

Several study groups now advocate the integration of circulating tumour DNA (ctDNA) for response evaluations as part of RECIST (Liquid Biopsy Response Evaluation Criteria in Solid Tumours) [3–5] since recent evidence suggests that ctDNA levels broadly correlate with tumour burden and proliferation status [6–9]. Consequently, ctDNA can serve as an indicator of evolving tumour burden and longitudinal monitoring of changes in ctDNA levels is emerging as an early signal of investigational drug activity in clinical trials [10–14].

Therefore, a systematic implementation of ctDNA kinetics in Phase I clinical drug trials could represent a rapid and dynamic orthogonal method to complement radiological imaging as well as PK/PD modelling in early clinical trials to identify early biological activity and further optimize dose selection [13, 15]. Integrating ctDNA into in phase 1 trials requires balancing costs, turnaround times, and the reliability of tumour fraction (TF) assessments. In most studies, the assessment of TF in ctDNA relies on mutation analysis. Beyond mutations, aneuploidy, somatic copy number alterations (SCNA), and ctDNA methylation patterns are also used to determine TF and predict clinical outcomes [16–19]. More recently, ctDNA fragmentomics features have gained attention as a tool for refining ctDNA response criteria [20, 21]. In addition to identifying the most cost-effective means to measure tumour fractions for an early clinical trial framework, another key question is to what extent a reduction in ctDNA levels during early treatment correlates with biological activity and molecular response.

In this perspective review, we will explore the opportunities and challenges to apply ctDNA approaches for both patient inclusion and monitoring during early clinical trials. This will enable a more informed data-driven decision process for patient selection, dose optimization as well as proof-of-mechanism readouts of drug biological activity and thus support the evolving paradigm in oncology early drug development. Finally, we focus on the use of cost-effective agnostic genome-wide (low-pass whole genome sequencing, fragmentomics) and methylation-based methods to permit a more systematic implementation of ctDNA in the Phase I clinical trial settings.

## Paradigm change in Phase I investigational drug trials

### I. Dose optimization rather than maximal tolerated dose

**Fig. 1:**
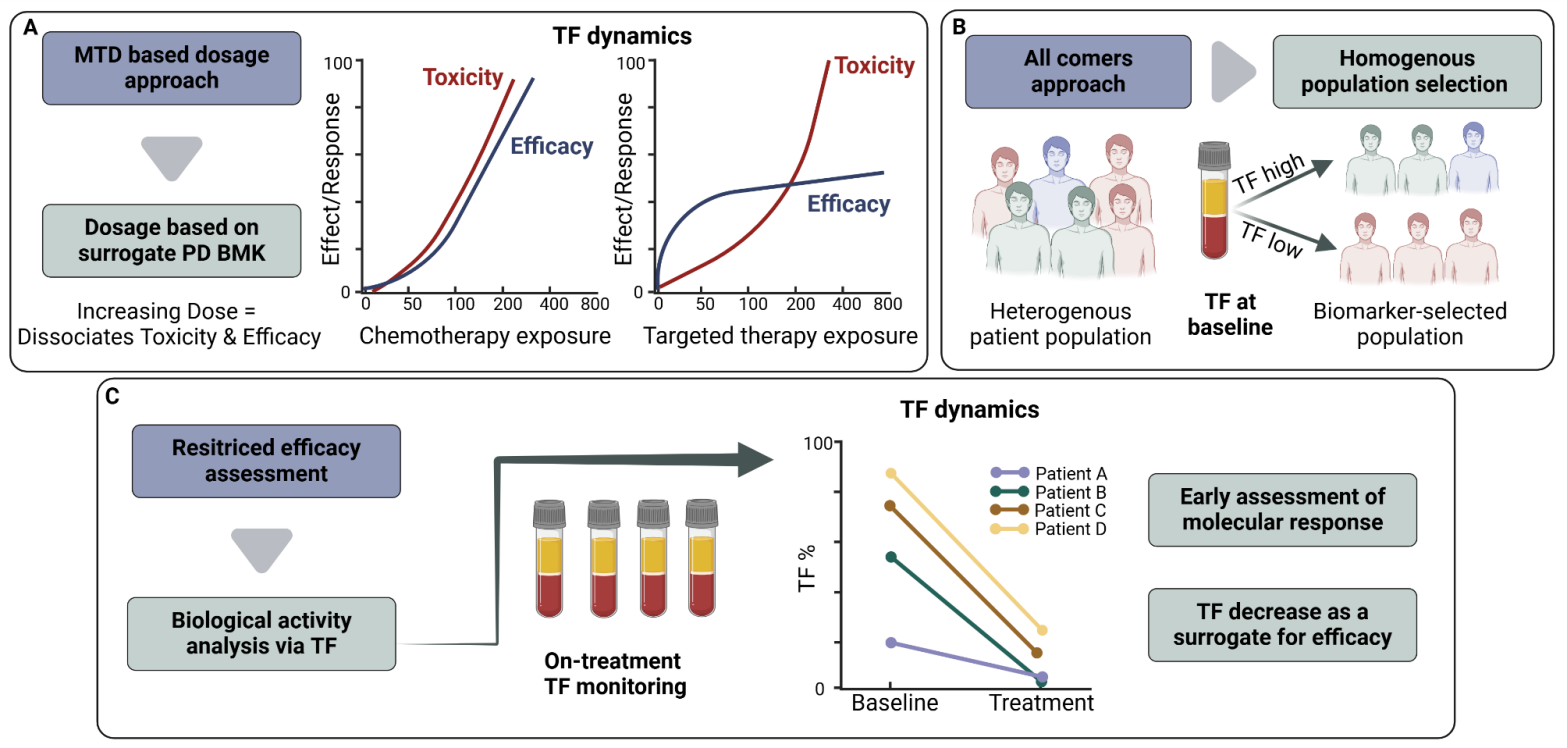
ctDNA baseline levels and kinetics as a supporting tool in the evolving paradigm of Phase I clinical drug trials. **A**) From Maximal Tolerated Dose (MTD)-based dosing to biomarker-driven optimization. Traditional chemotherapy schedules rely on escalating doses until reaching the MTD. However, many targeted therapies and immunotherapies achieve optimal target engagement at lower doses. Incorporating biomarkers - such as ctDNA tumour fraction (TF) dynamics - into early-phase studies can guide more precise dose selection, reduce unnecessary toxicity, and help identify the optimal therapeutic window. (Adapted from fda.gov) **B**) From “all-comers” to enriched patient populations. Assessment of baseline TF helps refine patient selection by reducing patient heterogeneity. This enrichment approach increases the likelihood of therapeutic response and supports earlier, more ethically sound access to investigational treatments. **C**) From limited efficacy assessments to early measures of biological activity. On-treatment TF monitoring provides an early indicator of molecular response, with decreases in TF levels serving as potential surrogates for clinical efficacy. PD BMK, biomarkers of pharmacodynamics; WES, whole exome sequencing; TOO, tissue of origin; Created in BioRender. Heitzer, E. (2024) https://BioRender.com/x33p735

Identifying the potential of new treatments in oncology Phase I trials (either as monotherapy or in combination with Standard-of-Care and/or other Investigational Products) by addressing the optimal drug dosage and schedule is key to expedite drug development and discern the optimal therapeutic window. Phase I study designs in oncology, still largely based on chemotherapy schedules, generally proceed via a defined dose escalation phase to determine a Maximum Tolerated Dose (MTD) [1] and based on the principal that an increasing dose will lead to increased tumour killing (Fig. 1A). However, in contemporary drug development this paradigm is no longer fully applicable to molecular targeted therapies (MTTs) and immunotherapies since they have target saturation limits below the MTDs suggesting that these therapies could be administered at lower doses with similar efficacy and fewer side effects. The need to re-visit dose-optimization was addressed in a recent review [22], showing examples of drugs, that following approval required modified doses and schedules for safety or tolerability, emphasizing the need to better address this question earlier in the process of drug development. During dose escalation, the use of PD biomarkers are key elements to support better pharmacokinetics (PK)/PD characterization and dose optimization. In this regard, Phase 1 trials should include adequate PK sampling and when feasible, PD sampling should be incorporated to determine the drug exposure that results in inhibition of the drug target and preliminary characterization of dose-exposure relationships. In addition, the use of ‘backfilling’ cohorts in Phase I dose-escalation studies enables the collection of additional information on the safety profile, optimal dose, pharmacokinetics and molecular activity of the investigational drug. To mitigate many of the failings regarding optimal doing, the US Food and Drug Administration (FDA) has published its opinion on the inadequate characterization of doses and schedules of oncology drugs. The FDA Project Optimus, which focuses on new oncology drug dose optimization, highlights the need for a more thorough evaluation of the optimal risk-benefit ratio prior to registration trials [23].

### II. Patients: from an “all-comers” approach to a more “targeted” population

Patients recruited into Phase I oncology drug trials for solid tumours are most often heterogeneous in terms of cancer type with advanced or oligometastatic progressive disease (Fig. 1B). These patients have generally failed multiple previous lines of treatment from Standard-of-Care to alternative lines of approved and/or investigational products. In addition, the patients´ tumours may have developed multiple resistant mechanisms and/or have become refractory to treatment. Historically, with cytotoxic chemotherapy and classical Phase I trial design, reported clinical outcomes such as low response rates, have brought into question the therapeutic appeal and ethical justification of Phase I trial enrolment. More recent real-world data studies on Phase I trials with targeted therapies and immunotherapy with an enrichment design have indicated more encouraging response rates [24–26]. The new evolving paradigm in Phase I clinical trials towards an enrichment design requires that we shift from an “all-comers” approach to a more “selected” patient population. This would require a more biomarker-driven population, with less heterogeneity in terms of cancer types and disease burden and consequently a higher likelihood for patients to respond to treatment. Indeed, one of the benefits of this evolving paradigm from an ethical perspective is that in this setting, the patient could have early access to an investigational drug (or combination thereof), with a higher potential to demonstrate response. This could enable an increased recruitment rate, as oncologists may be less reticent about referring patients to phase 1 drug trials with a therapeutic intent rather than just designed for safety & tolerability.

### III. Getting early signals of molecular response

The capacity to demonstrate early signals of clinical and molecular response of an investigational drug in Phase I could be a key driver to enable accelerated drug trials and reduce attrition in later phases. However, demonstration of clinical response in Phase I is extremely challenging as patients are recruited with a significant disease burden and heterogeneity. Moreover, the investigational drug is not administered at an optimal dose and/or schedule nor with the projected combination strategy. Currently, radiological imaging (RECIST & iRECIST) remains the mainstay for assessing clinical benefit in early clinical development trials [27, 28]. However, in the case of RECIST, the assessment of treatment response depends primarily on dynamic changes in gross macroscopic tumour volume in pre-selected target lesions, and this may fail to detect smaller changes in tumour burden more globally in different tumour sites. Considering the difficulties in assessing clinical response in Phase I, the need to seek alternative readouts of activity of the drug, such as molecular response, is warranted. In this regard, the use of tissue-based PD biomarkers, to enable Proof-of-Mechanism (PoM) and Proof-of-Principle (PoP) that demonstrate target engagement and show that the drug influences the expected signalling pathway, are critical to identify surrogates of biological activity. A promising alternative to tissue-based approaches is the use of ctDNA (Fig. 1C).

## Systematic implementation of liquid biopsy to inform the new paradigm

A potential use case of ctDNA assessments for enhancing decision-making and accelerating drug development in Phase I oncology trials is patient stratification and enrichment to identify patient subgroups with specific tumour fraction (TF) thresholds, improving trial recruitment efficiency and outcome predictability. There are multiple benefits associated with the assessment of tumour fraction at baseline before recruiting a patient to a Phase I trial including (i) identifying patients with varying risks of recurrence/progression, which enables to integrate TF as a confounding factor in retrospective data analysis or prospective patient selection based on TF-related risk of recurrence/progression, (ii) optimisation of clinical studies design by reducing the overall number of trial participants needed (reduce time and cost of studies), and (iii) improved understanding of survival endpoints via retrospective analysis of ctDNA levels and their prognostic value. The potential benefit of assessing ctDNA levels at baseline in early clinical development as an approach to assess therapeutic outcomes has been shown in many early clinical development studies following both chemotherapy and targeted therapies [29–31], and particularly in patients treated with immune checkpoint inhibitors [7, 32–36]. The assessment of the TF at inclusion in the clinical trial [37], can reflect disease aggressiveness with regards to the proliferation and overall tumour burden, will allow correlative analysis between TF and potential response or duration of response. A recent prospective analysis confirmed that high TF was associated with significantly worse overall survival (OS) and is thus a strong prognostic factor in patients with advanced solid tumours and represented a helpful tool in the process of patient selection for Phase I trial entry [38].

Another promising approach is the provision of real-time insights into treatment response, enabling adaptive trial designs and early go/no-go decisions. Changes in TF may indicate early molecular responses to investigational drugs, even before imaging or clinical outcomes are evident. In a recent study, Sanz-Garcia and colleagues demonstrated that a decrease in ctDNA within the first 4 weeks of investigational IO therapy was associated with improved treatment outcomes, with a more marked effect when a decrease in ctDNA of > 50% from baseline was observed [39].

## Systematic implementation of ctDNA at baseline and during treatment

### I. challenges of mutation-based assessment of TF and kinetics

**Fig. 2:**
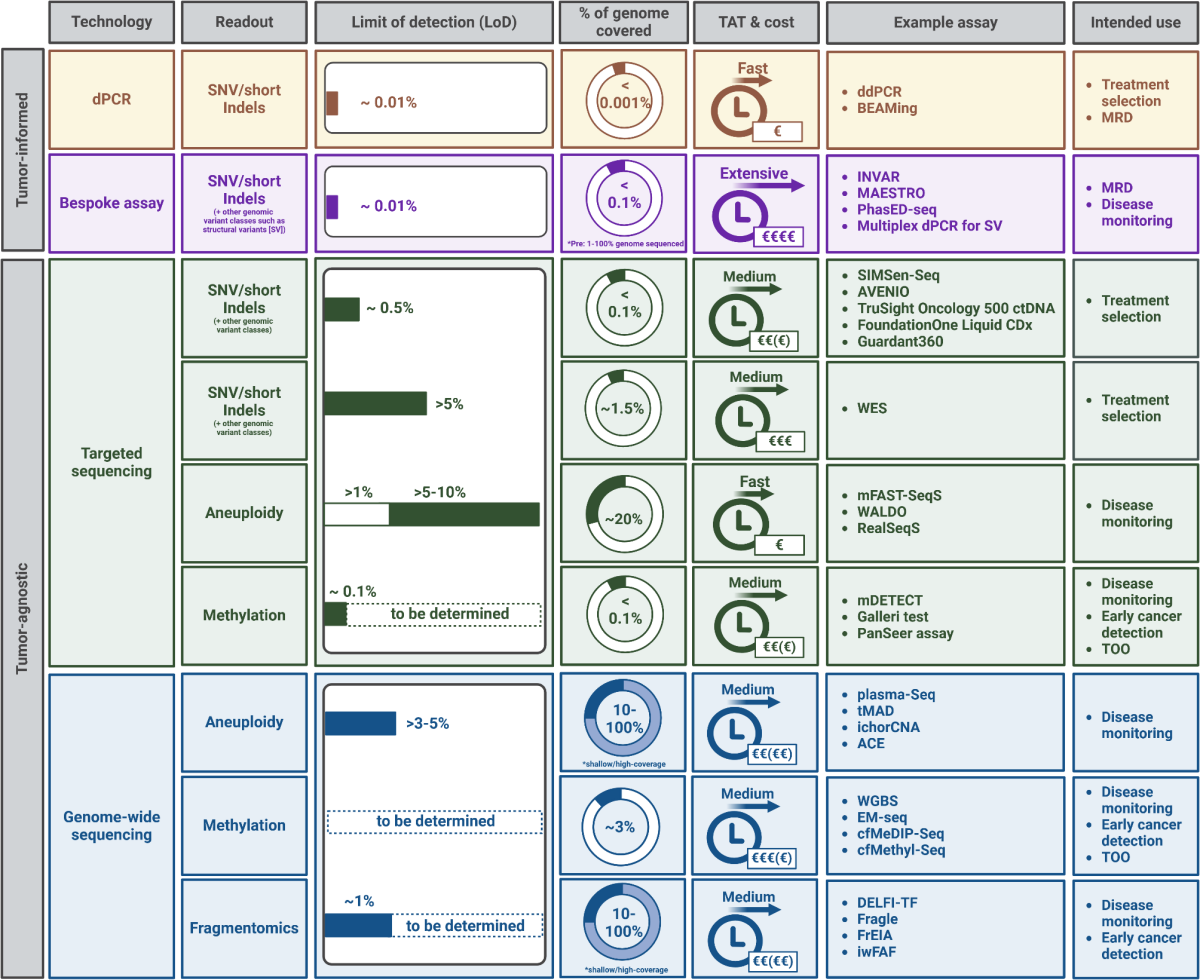
Plethora of ctDNA-based approaches. The diagram highlights a selection of tumor-informed and tumor-agnostic methodologies designed to analyze single nucleotide variants (SNVs), aneuploidy, methylation patterns, and fragmentomic features. Choosing the right ctDNA assay depends on several factors, including the limit of detection (LoD), genome coverage, turnaround time (TAT), and cost. Shallow whole-genome sequencing is often adequate for aneuploidy- and fragmentomics-based approaches, whereas high-coverage datasets may be used for greater detail but come with increased computational demands and costs. For each technology, examples of assays and their primary applications are provided. Created in BioRender. Heitzer, E. (2024) https://BioRender.com/c35w276

In general, TF in cell-free DNA (cfDNA) is defined as the fractional proportion of tumour DNA relative to total cfDNA. Even in highly advanced patients, TF are highly variable and can be influenced by multiple factors [40] such as overall tumour burden, and disease activity (progressing, stable or responding to systemic therapy), patient-context factors such as fasting status or physical activity prior to blood collection. Moreover, technical pre-analytic factors related to sample acquisition, transport, and sample processing procedures can confound the true representation of tumour derived DNA. Furthermore, TF calculated from mutations - reflected as the variant allele frequency (VAF) - are influenced by factors like copy number alterations, loss of heterozygosity, tumour ploidy, and clonal diversity within ctDNA. Advanced tumours show a high degree of spatial and temporal heterogeneity manifested by polyclonal properties. Therefore, highly sensitive tumour-informed approaches using bespoke assays do not reflect the genetic landscape of the disease [41] and tumour-agnostic approaches that require no prior knowledge about the tissue are preferable in a Phase I setting (Fig. 2). Since most gene panels are designed for target detection rather than ctDNA tracking, they have limitations in detection sensitivity and may not effectively reveal emerging subclones. Larger panels capable of aggregating multiple mutations per patient offer potential for high sensitivity ctDNA tracking but are still quite costly for repeated analysis [6]. Chemotherapy and immune infiltration can also affect clonal composition, potentially inflating VAFs of subclonal mutations, making them unreliable for reflecting the true tumour burden. While using mean VAF can mitigate sampling bias and temporal variability, the maximal VAF better correlates with aneuploidy-based TF measures [42]. Moreover, VAFs are affected by biological noise from clonal haematopoiesis, necessitating additional sequencing of peripheral blood leukocytes, which increases costs [43]. Non-tumour cfDNA variability, influenced by factors like exercise or infection, can artificially alter VAFs, prompting the use of concentration-based metrics (e.g., mutated fragments per millilitre of plasma). However, recent analysis suggests concentration-based metrics may be more prone to certain technical biases, such as coverage bias [44].

Another important question is how to measure ctDNA kinetics and molecular response [3–5]. To assess changes of ctDNA over time several measures based on mutations were described in the literature, with both absolute and relative changes widely applied in an attempt to define a molecular ctDNA response [4, 15]. The easiest way of measuring response is a binary read out by assessing ctDNA clearance, i.e. the decrease of positive ctDNA results to a reduction below the limit of detection (LoD) [33, 45, 46]. However, since response might not always be associated with ctDNA clearance, this method might not consider patients with significant decreases as responders. Therefore, many study groups calculate relative changes between baseline and an on-treatment time point (delta) to assess molecular response. However, a relative change does not consider the overall levels of ctDNA and may therefore be misleading, which is why proportional changes, percent changes or ratios may better reflect the actual tumour burden [47–50]. Studies by Zhang et al. and Thompson et al. have shown that the ratio of the mean VAF best predict OS and PFS [48, 49].

Other studies reported mutant allele counts or frequencies directly, but there is a growing need for normalized scores to quantify ctDNA kinetics. Kato et al. proposed Mutation Allele Ratio in Therapy (MART), i.e. the ratio of plasma mutation scores as a normalized scores to quantify ctDNA kinetics [51]. Similarly, the Circulating DNA Ratio (CDR) score, also known as the molecular response ratio, compares VAFs during therapy to baseline.

### II. Aneuploidy-based assessment of tumour fractions and kinetics as a cost-effective alternative to mutations

In early Phase I drug trials there is usually no critical need to obtain a full molecular portrait of the tumour but rather to have an unbiased measure of the TF to assess signals of response/efficacy. Aneuploidy-based assessments of TF that enable a quantification of ctDNA levels beyond evaluation of mutation allelic fraction, offer a potentially important and significantly under-exploited opportunity to further develop more systematic evaluation of ctDNA kinetics in clinical practice [52] and in early clinical drug development [13, 39].

Such methods come with a short turn-around-time and may be a cost-effective alternative to mutation-based approaches. Since it is considered that > 90% of solid tumours are aneuploid and contain multiple SCNA [53], such methods would enable a broad patient coverage.

Amplicon-based methods using sequence-specific primers to enrich for uniquely mappable repetitive sequences have been proposed for this purpose [17, 54–56]. Such protocols offer many advantages including a very fast and simple workflow, a minimum requirement for input DNA (5pg-1ng), reduced sequencing costs and a simplified computational analysis. An extremely simple and low-cost method for estimating TF represents the modified Fast Aneuploidy Screening Test-Sequencing System of LINE-1 sequences (mFAST-SeqS) method [17]– originally developed to detect trisomy 21 in fetal cfDNA [56]. Chromosome-arm wide read count deviations of repetitive elements summed up to a genome-wide z-score are used as TF surrogates with a limit of quantification (LoQ) of 5–10% TF depending on the number and level of SCNA. Despite this limited sensitivity, changing genome-wide mFAST-SeqS z-scores provide early means of treatment response and are closely correlated to ichorCNA-derived TF [57]. Moreover, longitudinal trajectories of z-scores as surrogates for TF can predict risk of progression in metastatic breast cancer patients undergoing CDK4/6 treatment [58]. A more recent study showed that mFAST-SeqS aneuploidy scores measured prior to treatment with pembrolizumab in metastatic urothelial cancer, significantly correlated with ctDNA levels measured by an orthogonal approach and were independently associated with lack of clinical benefit [59].

Efforts were being made to push the sensitivity of aneuploidy-based assays leveraging repetitive elements such as Within-Sample AneupLoidy DetectiOn (WALDO) [54] or Repetitive Element AneupLoidy Sequencing System (RealSeqS) [55], but none of them were used for monitoring purposes yet. Most of these amplicon-based methods do not directly infer the TF but rather calculate the deviation in read counts to a control population. For this reason, the TF surrogate values are biased towards the degree of aneuploidy in a sample, i.e. samples containing more aneuploidy will provide higher values and may be easier to detect at a given neoplastic cell content compared to samples with a low degree of genetic instability. However, intra-patient variations of tumour levels are not affected by this issue and several studies have demonstrated utility for response monitoring in advanced cancer patients [57].

Low-pass whole-genome sequencing (LP-WGS) offers another cost-effective and broadly applicable approach for estimation of TF. Early studies used read count based genome-wide z-scores as an estimation of TF by comparing global copy number alterations in a given plasma sample to a panel of normal healthy donors (plasma-Seq) [60] or scores like the trimmed median absolute deviation (tMAD) from the copy number neutral state [61] as TF surrogates. More recently, computational models such as ichorCNA [16] and ACE [62] considering tumour purity, ploidy, and SCNA were developed to estimate ctDNA TF. For example, ichorCNA uses a probabilistic model, implemented as a hidden Markov model to simultaneously segment the genome, predict large-scale copy number alterations, and estimate the TF of a LP-WGS sample. Several studies have demonstrated the utility of LP-WGS for monitoring purposes in the advanced setting [30, 57, 63–65]. In a small cohort of patients, Moser et al. conducted a comparative analysis of TF estimations with plasma-Seq and ichorCNA (limit of detection - LoD, 3%) and NGS-panel based sequencing (0.1-1%) showing similar changes and trends in ctDNA kinetics to determine an early response to chemotherapy [30]. In a prospectively enrolled, advanced stage, pan-cancer cohort with different advanced solid tumours and using LP-WGS ctDNA-based monitoring, the degree of ctDNA reduction was shown to strongly correlate with long-term outcome using LP-WGS ctDNA-based monitoring [64]. The current LoQ for these methods is a TF of 3% with a LoD of 1% [16], which - given the current known range for baseline TF in various advanced cancers - may not sufficiently sensitive to reliable determine TF in all patients [37, 42]. However, in the context of an early clinical trial setting, where the majority of patients are highly advanced and have progressive disease, LP-WGS would provide a rapid and affordable approach to assess TF. An extensive real-world evidence study showed that over 94% (*n* = 22,130) of patient samples had detectable ctDNA, with a median TF of 2.2%, varying between 17.7% and 1.1% depending on the cancer type [42]. An earlier report [66] showed similar trends in ctDNA fractions with a large pan-cancer data set (*n* = 21,807). In a more recent study, an outcome analysis study of 1725 advanced/metastatic patient plasma samples showed varying median TF values for metastatic castration-resistant prostate cancer (13,3%) metastatic breast cancer (4.1%) advanced non-small-cell lung cancer (2.1%), or metastatic colorectal cancer (7.8%) [37]. Interesting, a study adopting a LP-WGS/ichorCNA methodology, from 246 plasma samples with advanced and metastatic mBC patients, 178 (72.4%) had TF of ≥ 3% (range 4–84%; median 9.4%) [67].

### III. Non-genetic features as surrogates for TF

Aberrant methylation is a hallmark of cancer, including global hypomethylation and promoter-specific hypermethylation and several studies have demonstrated that these changes are not only diagnostic but also indicative of tumour burden [68–70]. Recently, it has been shown that TF in cfDNA can be deciphered from methylation signatures [18] and there is a growing interest in ctDNA methylation as an additional variable for developing molecular response criteria [19, 71].

Technological advancements have enabled the detection of methylation in ctDNA using techniques such as targeted bisulfite sequencing [72, 73], methylation-specific PCR [74, 75], and newer approaches like cfMeDIP-seq (cell-free methylated DNA immunoprecipitation sequencing) [76]. These methods allow for sensitive detection of cancer-specific methylation sites, correlating with tumour burden in advanced cancer patients.

Epigenetic-based TF estimation offers several advantages over mutation-based methods. Unlike mutations, which may be absent or rare in specific tumour types, methylation changes are nearly universal in cancer and often occur early in tumorigenesis. Additionally, methylation markers are less affected by clonal hematopoiesis or other sources of biological noise that can confound mutation-based analyses.

Evidence suggests that changes in a small number of cancer-specific DNA methylation marks correlate with clinical outcomes and can predict therapeutic benefits [77, 78]. In hepatocellular carcinoma, triple-negative breast cancer, and colorectal cancer (CRC) methylation quantification in specific amplicons enriching for differentially methylated CpGs has effectively monitored tumour burden [69, 72, 79]. Longitudinal assessment of cfDNA methylation using an epigenome-wide array in metastatic prostate cancer patients also unveiled dynamic patterns associated with disease progression and therapy administration [80]. A cost-effective genome-wide approach is cfMeDIP-seq, that was recently shown to strongly correlate with OS and PFS, representing a promising plasma-based predictive epigenetic biomarker in patients treated with checkpoint blockade [81].

Another promising application is the ability to estimate the tissue of origin (TOO) of ctDNA through blended methylation signatures. Shifts in the composition of TOO may also be used for monitoring purposes. To this end, reference-based deconvolution has been most widely adopted methodology in previous studies [82], but evidence with respect to assessing treatment response is currently not available.

The analysis of biophysical properties of ctDNA, including fragment size distribution, end motifs, and chromatin-associated patterns may also offer a complementary and highly sensitive method for monitoring disease dynamics in advanced cancers [83]. Although these technologies are still in their infancy and were mostly developed for classification purposes enabling early cancer detection, some initial data show utility for monitoring. DEFLI (DNA evaluation of fragments for early interception) was originally developed as a cancer screening tool [20], but more recently this machine learning model incorporating genome-wide fragmentation features was also used to estimate cfDNA tumour burden for treatment response monitoring and clinical outcome prediction in metastatic CRC. DELFI-TF strongly correlated with standard VAF testing based on ddPCR pointing out to a high sensitivity of this approach [84]. Similarly, Renaud and colleagues proposed non-negative matrix factorization of fragment length distributions as a novel and completely unsupervised method for studying fragment length patterns in cfDNA that enables a simultaneous estimate of fragment length signatures and their weights in each sample. The weights of this signature correlated strongly with ctDNA levels - nearly as good as ichorCNA - without using any information about variants or ctDNA levels [85]. Fragle, is another multi-stage machine learning model that quantifies ctDNA levels directly from a cfDNA fragment length density distribution from LP-WGS data. Preliminary data demonstrated a superior limit of detection for Fragle compared to ichorCNA for both baseline (100% versus 53%) and during treatment (100% versus 41%). Budhraja and colleagues took a different approach and developed a metric based on genome-wide differences in fragment positioning, weighted by fragment length and GC-content (information-weighted fraction of aberrant fragments (iwFAF), which strongly correlated with TF [21].

Multi-modal approaches, i.e. the integration of genomic alterations, methylation patterns, and fragmentomics may further enhance the detection and monitoring of cancer and more accurately represent ctDNA dynamics compared to using a single biomarker [86–89]. For example, Stutheit-Zhao and colleagues developed a pan-cancer methylation signature to quantify cancer-specific methylation (CSM) and fragment-length score (FLS) that predict overall survival and progression-free survival in patients treated with pembrolizumab, independent of tumor type [76].

Despite their promises, epigenetic or fragmentomics-based assessment of ctDNA TF faces challenges. The cost and complexity of epigenetic assays remain barriers to widespread adoption. Moreover, reference-based deconvolution methods for TOO estimation are still in early stages for assessing treatment response. Furthermore, since most of these technologies rely on machine learning approaches, variability in sample preparation, sequencing depth, and data interpretation can impact reproducibility. Therefore, extensive validation and standardizing of these processes is essential for clinical implementation.

## Conclusion

A systematic implementation of TF assessments, in Phase I clinical drug trials represents a great opportunity to improve decision-making in early drug development by enabling not only patient selection and dose optimization but also proof-of-mechanism readouts of drug biological activity. To this end, rapid and cost-effective methods, without a necessity of prior knowledge of the genetic composition of tissues, and that enable an accurate assessment of TF before and during treatment fulfil this purpose. While aneuploidy-based methods meet these criteria in terms of costs and turn-around time, the trade-off is evidently a loss of sensitivity. Nevertheless, given our improved understanding of the extent of TF variation within and between different cancer types, a large proportion of patients in Phase I clinical drug trials would have detectable TF with the current aneuploidy-based methods. Moreover, in the context of Phase I clinical drug trial development with advanced metastatic disease, sensitivity is less an issue and where the main objective is to assess FT levels at baseline as well as changes from baseline under treatment. Further prospectives studies will be required to determine what level of TF at baseline and kinetic decrease are key decision enablers for early phase 1 trials.

## Data Availability

Not applicable.

## Abbreviations

cfDNA: Cell free DNA
CRC: Colorectal cancer
ctDNA: Circulating tumour DNA
DEFLI: DNA evaluation of fragments for early interception
FDA: Food and Drug Administration
LoD: Limit of detection
LoQ: Limit of Quantification
LP-WGS: Low-pass whole-genome sequencing
mFAST-SeqS: Modified Fast Aneuploidy Screening Test-Sequencing System of LINE-1 sequences
MTD: Maximum Tolerated Dose
MTT: Molecular targeted therapies
PD: Pharmacodynamics
PK: Pharmacokinetics
RECIST: Response Evaluation Criteria in Solid Tumours
SCNA: Somatic copy number alterations
tMAD: Trimmed median absolute deviation
TOO: Tissue of origin
TF: Tumour fraction
VAF: Variant allele frequency
